# Sciatic Nerve and Its Anatomical Variations: In-Depth Understanding Acquired During Dissection Classes

**DOI:** 10.7759/cureus.60083

**Published:** 2024-05-11

**Authors:** Jacopo Junio Valerio Branca, Giulia Guarnieri, Annamaria Morelli, Carlo Benedini, Niccolò Fagni, Massimo Gulisano, Alessandra Pacini, Ferdinando Paternostro

**Affiliations:** 1 Experimental and Clinical Medicine, University of Firenze, Firenze, ITA; 2 Physical Medicine and Rehabilitation, ICLO Teaching and Research Center, Verona, ITA; 3 Otorinolaringoiatry, Azienda Ospedaliero-Universitaria Senese (UOSA), Siena, ITA

**Keywords:** anatomical variations, piriformis syndrome, piriformis muscle, sciatic nerve, ischiatic nerve

## Abstract

Knowledge of anatomical variability is extremely important in order to better understand the etiology of pain, if present, or to avoid iatrogenic consequences. Sometimes the anatomical "anomalies" have the same anamnesis but different causes. For example, sciatic neuralgia may be caused by a herniated disc or it may have a different origin. The sciatic nerve (SN), also known as the ischial nerve, is the widest in the human body. This huge peripheral nerve originates from the roots of the lumbosacral plexus (L4-S3) and passes through the great sciatic foramen, under the piriformis muscle (PM). However, there is much variability in the pattern of SNs about the muscle, which has been known since the first half of the 20th century. In the present study, we describe six different case reports of anatomical variations of the SN and its interplay with the PM. The observations were made during dissection classes at the ICLO Teaching and Research Centre (Verona, Italy), on both male and female cadavers aged between 58 and 84 years. The SN was reported as a single and divided nerve into the tibial nerve (TN) and the common peroneal nerve (CPN), passing alone above, below, or between the PM. However, the two parts of the SN may also interact with the PM in different ways, adding to the anatomical variability. A thorough knowledge of the anatomical variations in any part of the human body is extremely important. The various techniques used, from imaging to autopsy or surgery, are also useful in the SN pathway. Thus, the anatomical features and the understanding of each variation are useful for a correct approach that can lead to an effective and correct treatment with a favorable outcome.

## Introduction

In recent years, knowledge of anatomical variations has become an important skill not only in surgery but also in health care. A look at the PubMed database shows that the number of articles containing the term "anatomical variations" increased steadily in the second half of the 20th century [1]. Indeed, even if the first article on this topic was published in 1898 by Cunningham, the increasing number of articles was only reported from the 60s onward and only exceeded a thousand units in 2011 [2].

Such strange evidence could be mainly due to the increasing technologies and instruments to evaluate anatomical variability [1]. However, the importance of knowing the anatomical variations in clinical practice should also be taken into account. These differences are not only important to better understand how pathologies can occur but also to avoid critical and adverse effects during surgery or other therapies.

The anatomical variations can cover a wide range of body regions and anatomical structures, ranging from vasculature to organs located in different body regions. Nowadays, due to an increase in anatomical variability knowledge, it is worth noticing that “anormalities” might be considered an evolutionary leap [3]. Indeed, the "normal" anatomical variations are often invisible because they do not cause pain or difficulty [4]. Thus, anatomical variations are defined as a different arrangement of an anatomical structure without causing a demonstrable impairment of its function [5].

However, considering that abnormalities, impairments, or pain may be present, it is necessary to have an in-depth knowledge of the common and standard anatomy, but always keep in mind the possible variability in order to reduce the iatrogenic incidence and injuries [4,6,7]. Such an extremely important background should also be combined with an understanding of the close relationship of organs or tissues that may be altered. In this respect, sciatic neuralgia, commonly known as sciatica, is a good example.

Sciatica is a clinical term first used in 1934 by Mixter and Barr and is mainly caused by a herniated intervertebral disc that presses on the sciatic nerve (SN), although there may be other causes [8,9]. Such impairment is not only focused on pain but also changes in the biomechanics of the pelvic girdle are evident, leading to physical inability to move and a reduction in the patient's quality of life [10].

The sciatica is mainly resolved by hernia repair, but the symptom of sciatica pain may be due to other causes, commonly known as nondiscogenic sciatica or extraspinal sciatica [11,12]. Thus, the main cause of this impairment is due to the close relationship between the SN and the pyriformis muscle (PM), hence the name piriformis syndrome (PS), a term first used by Robinson in 1947, characterized by the entrapment of the SN within the muscle volumetry [13]. However, it is necessary to distinguish between two different types of PS: primary and secondary PS [14]. Clinicians should also pay attention to the PM, which may be inflamed, spastic, or have other indirect involvement such as hematoma, osteophytes, neoplastic mass, and others [15]. In both cases of PS, the clear and correct anatomy and its variability should be known.

The SN is the largest nerve in the human body and consists of two components, the tibial and the common peroneal [16]. The SN arises as a common nerve from the lumbosacral plexus. It leaves the pelvis through the greater sciatic foramen, usually below the PM, and descends between the greater trochanter of the femur and the ischial tuberosity of the pelvis, at the back of the thigh, dividing into two nerves, the tibial and the common peroneal (fibular), near the posterior surface of the knee joint, the popliteal fossa. Above, after the sciatic foramen, the SN lies deep to the gluteus maximus muscle, initially resting on the posterior ischial surface with the quadratus femoris nerve between them. It passes posterior to the obturator internus muscle to the S3 spinal nerves [17].

Due to its course and close relationship and interaction with the PM, the variations of this muscle, if present, should also be known [18]. The PM is an external muscle of the hip, located in the gluteal region, and together with other muscles allows different movements of the hip, such as lateral rotation, abduction, and extension. It forms a canal with the superior gluteal muscle, through which the SN passes, and this is the main reason why its anatomy is important for knowledge of the PS [19]. Regarding the anatomy of the PM, the proximal insertion of the muscle is on the anterior surface of the sacrum and the sacro-tuberous ligament, and the fibers are directed laterally to the superior greater trochanter of the femur, passing through the greater sciatic foramen, above the sacrospinous ligament [20]. It is also worth noting that the PM is close to other muscles in the same region, such as the gemelli muscles, the obturator internus, and the glutei medius and maximus. In fact, as clearly reported by Probst and colleagues, the variability of the PM should also be considered when looking at the diameter of the piriformis tendon, which ranges from 3 to 9 mm. Such a difference is mainly due to the variations of the PM insertion on the femur: indeed, the PM insertion tendon can be fused with the closest muscle, i.e., the gluteus medius, but also with the obturator internus together with the superior gemellus or the gluteus medius [21].

However, an interesting hypothesis as to why interactions between the SN and the PM may occur through an anatomical variation can be explained by focusing on the different embryological developmental timing. Indeed, the SN is formed at approximately six weeks, whereas the PM is formed at eight weeks of embryonic stage and the PM insertion muscle is definitive at approximately 15 weeks [22]. However, it has been reported that also genetic factors, together with embryological basis, may have a key role in the neuro-muscular variations in the gluteal area [23]. Taken together, the SN and PM interactions and anatomical variations were first described in 1937 by Beaton and Anson, who clearly drew the relationship that occurred within these two compartments [24].

In the present study, we try to highlight the importance of anatomical dissection for the future knowledge of clinicians, especially in relation to sciatica neuralgia, which is very common in the world population, and disc herniation is a less common cause of the disease. Three case reports were previously presented as a meeting abstract at the 76th National Congress of the Italian Society of Anatomy and Histology (SIAI - Società Italiana di Anatomia e Istologia), which was held in Modena, Italy, on 11-13 September 2023.

## Case presentation

Case findings

In the present study, the SN and its close interaction with the PM were analyzed and reported during different dissection classes. The anatomical variability of the nerve, consistent with that shown and classified by Beaton and Anson and others, was observed in six lower limbs of cadavers of both sexes, aged between 58 and 84 years [21,22,24,25]. The dissections were performed in an institute specialized for these purposes, the ICLO Teaching and Research Centre (Verona, Italy), by dissector technicians, clinicians, and professors, between January 2023 and April 2024, on a total of 50 lower limbs.

**Figure 1:**
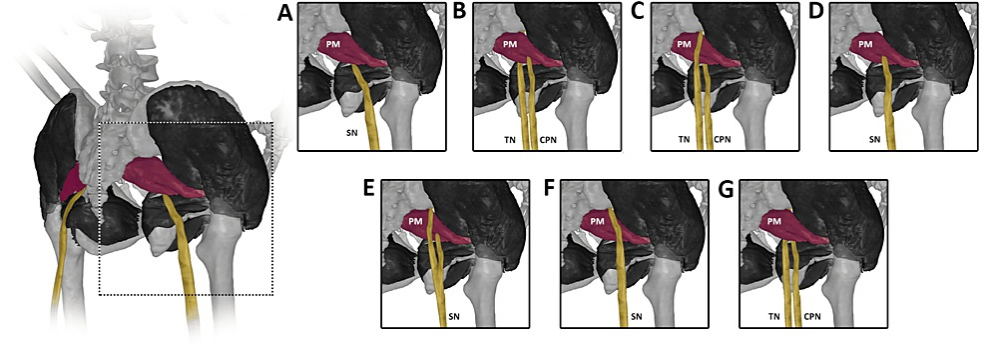
Different anatomical variations of the SN and its relationship with PM. (A) The “normal” most common SN path below the PM. (B) The divided SN into its CPN and TN components passing between and below the PM. (C) The CPN and TN components of the SN passing above and below the PM, respectively. (D) The undivided SN passes between the PM. (E) The divided SN passing above and between the muscles. (F) The undivided SN emerging above the PM. (G) Both the CPN and TN parts of the SN passing below the PM. PM, piriformis muscle; SN, sciatic nerve; TN, tibial nerve; CPN, common peroneal nerve Images created and modified from the SECTRA Education Portal, Dissector Atlas v. 6.3.5, epsectra.com. Dissector Atlas is powered by Virtual Human Dissector and provided by Touch of Life Technologies.

As clearly reported and documented, the most common relationship is shown when the undivided SN exits the great ischiatic foramen passing underneath the PM, as clearly shown in Figure 1A.

However, the SN has different "normalities" that are present in the world population. For example, the SN may pass above or between the PM. More interestingly, the SN can be divided into two components (the tibial nerve (TN) and the common peroneal nerve (CPN)), thus passing through and below the PM, CPN, and TN, respectively. On the other hand, the TN and CPN branches may pass above and between the PM or just above and below the PM. Finally, it should be noted that the divided SN may remain divided after passing the PM or it may reunite with the SN, as clearly reported in Figures 1B-1G [26].

**Figure 2:**
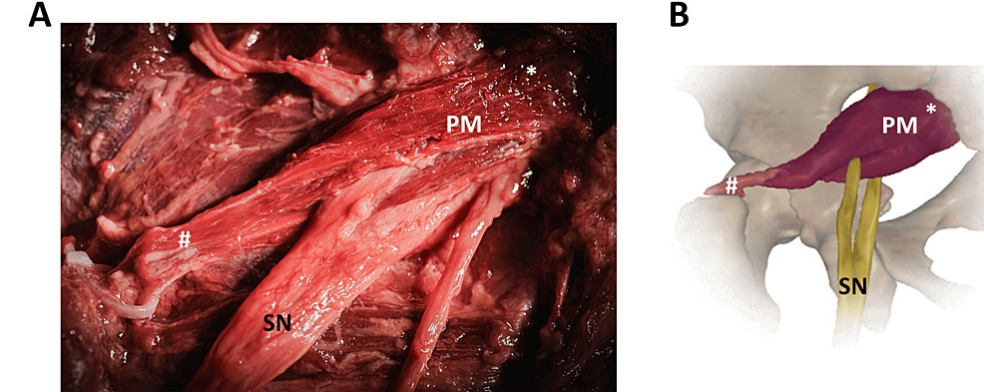
The SN division passing between and below the PM. ^*^origin, proximal insertion ^#^insertion, distal insertion (A) Anatomical variation observed in a 79-year-old male. (B) Representative picture of the SN variant and its relationship with PM. PM, piriformis muscle; SN, sciatic nerve The image in panel A was reprinted and modified from the book Anatomia Fotografica. Image credit: Carlo Benedini and Ferdinando Paternostro [27]. The image in panel B was created and modified from the SECTRA Education Portal, Dissector Atlas v. 6.3.5, epsectra.com. Dissector Atlas is powered by Virtual Human Dissector and provided by Touch of Life Technologies.

In Figure 2, the anatomical variation was observed in a 79-year-old male, reporting that the SN divided during its passage between and below the PM.

**Figure 3:**
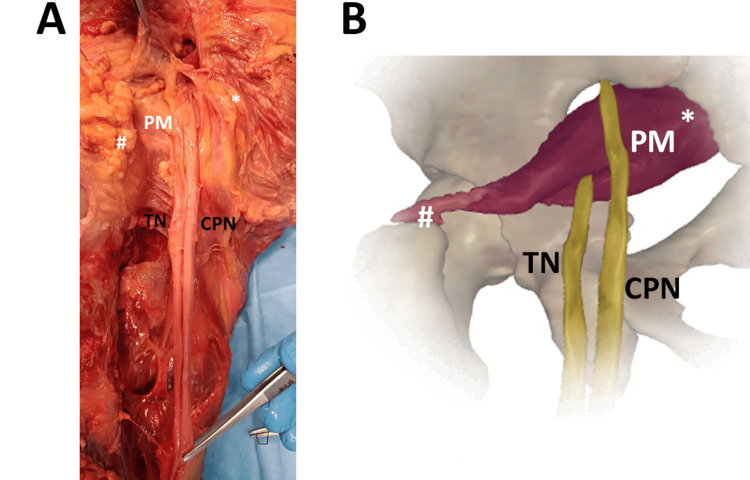
The SN division passing above and between the PM. ^*^origin, proximal insertion ^#^insertion, distal insertion (A) The real anatomical variation was observed in an 83-year-old female. The operator indicates the two SN components with tweezers. PM, piriformis muscle; TN, tibial nerve; CPN, common peroneal nerve (B) Image created and modified from the SECTRA Education Portal, Dissector Atlas v. 6.3.5, epsectra.com. Dissector Atlas is powered by Virtual Human Dissector and provided by Touch of Life Technologies, showing the SN division into TN and CPN and their relationship with PM.

During dissection classes, another interesting case was observed in an 83-year-old female, as reported in Figure 3. The dissected lower limb clearly showed that the SN divided into its two components, the TN and the CPN, passed between and above the PM, respectively.

**Figure 4:**
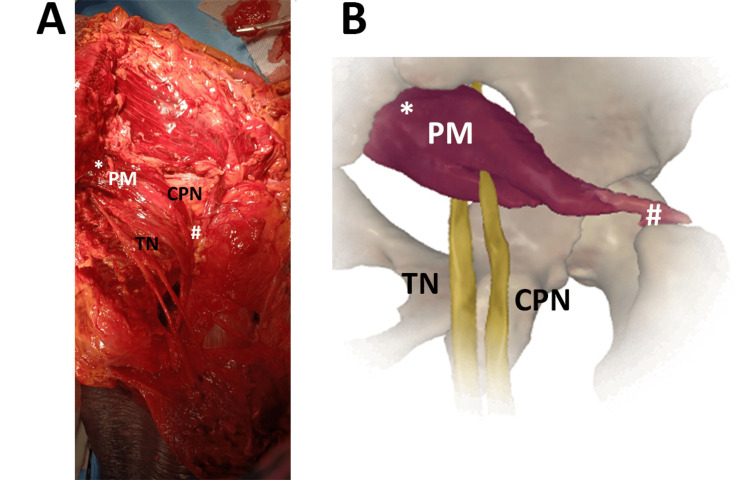
The SN division passing between and below the PM. ^*^origin, proximal insertion ^#^insertion, distal insertion (A) The SN anatomical variant was observed in an 81-year-old female. PM, piriformis muscle; TN, tibial nerve; CPN, common peroneal nerve (B) Image created and modified from the SECTRA Education Portal, Dissector Atlas v. 6.3.5, epsectra.com. Dissector Atlas is powered by Virtual Human Dissector and provided by Touch of Life Technologies, showing the relationship between the PM and the TN and CPN passing below and between the muscle, respectively.

In an 81-year-old female (Figure 4), the gluteal region of the external surface of the hip clearly shows the TN passing below the PM and the CPN emerging between the muscle.

**Figure 5:**
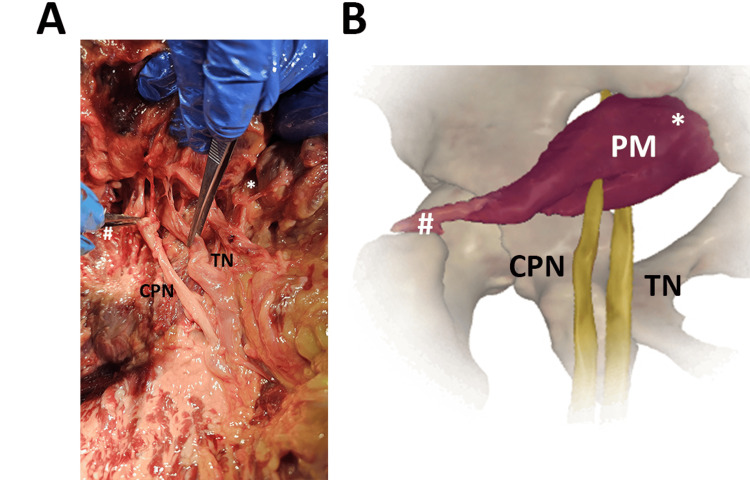
The CPN and TN components of the SN passing between and below the PM. ^*^origin, proximal insertion ^#^insertion, distal insertion (A) Representative real anatomical variant observed in a 76-year-old female. PM, piriformis muscle; TN, tibial nerve; CPN, common peroneal nerve (B) Image created and modified from the SECTRA Education Portal, Dissector Atlas v. 6.3.5, epsectra.com. Dissector Atlas is powered by Virtual Human Dissector and provided by Touch of Life Technologies, showing the relationship between the PM and the TN and CPN passing, respectively, below and between the muscle.

**Figure 6:**
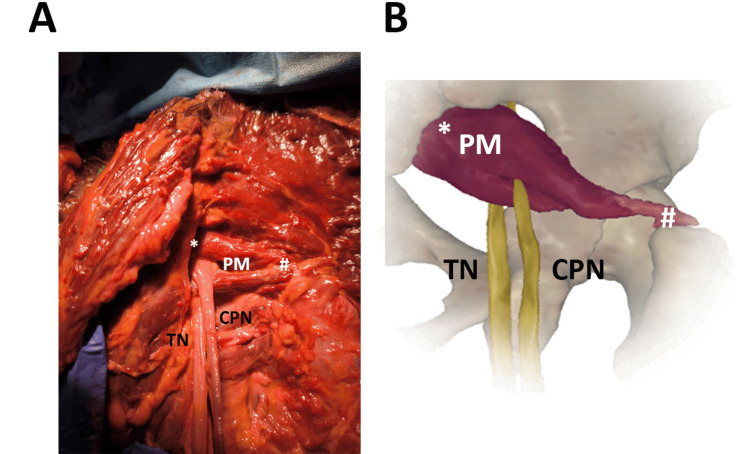
The CPN and TN components of the SN passing between and below the PM. ^*^origin, proximal insertion ^#^insertion, distal insertion (A) Representative real anatomical variant observed in a 58-year-old female. PM, piriformis muscle; TN, tibial nerve; CPN, common peroneal nerve (B) Image created and modified from the SECTRA Education Portal, Dissector Atlas v. 6.3.5, epsectra.com. Dissector Atlas is powered by Virtual Human Dissector and provided by Touch of Life Technologies, showing the relationship between the PM and the TN and CPN passing, respectively below and between the muscle.

**Figure 7:**
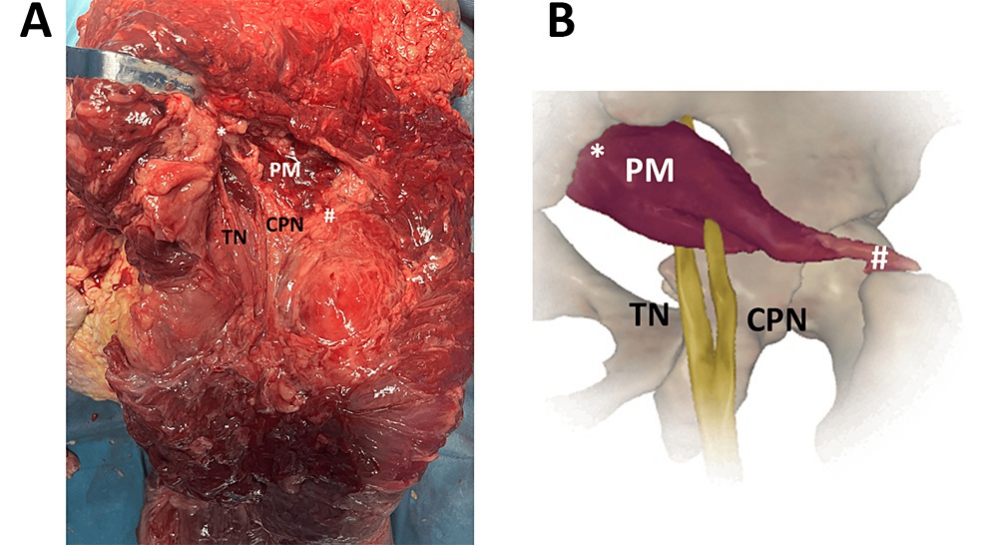
The CPN and TN components of the SN passing between and below the PM. ^*^origin, proximal insertion ^#^insertion, distal insertion (A) Representative real anatomical variant observed in a 76-year-old female. PM, piriformis muscle; TN, tibial nerve; CPN, common peroneal nerve (B) Image created and modified from the SECTRA Education Portal, Dissector Atlas v. 6.3.5, epsectra.com. Dissector Atlas is powered by Virtual Human Dissector and provided by Touch of Life Technologies, showing the relationship between the PM and the TN and CPN passing, respectively, below and between the muscle.

Finally, the last three cases were found in females, respectively, 76-, 58-, and 76-year-old. As reported in Figures 5-7, the PM clearly split the SN into the CPN and TN, passing between and under the muscle.

Dissection technique

The SN observation was performed by careful dissection of the gluteal region of the various bodies, following anatomical dissection techniques on cadavers. The inclusion criteria were that the lower limb should be in optimal condition and well-preserved to allow correct dissection and photography. It was also recommended that the cadavers be fresh and not fixed. The room temperature was maintained at 18-20°C and ventilation was constant.

Dissection was carried out with the cadavers in a prone position on the dissection table, exposing the gluteal region and posterior thigh. Two operators were present per cadaver, each with a 22-blade scalpel and surgical forceps, to proceed with the dissection.

The first operator used the scalpel blade to cut through the skin and subcutaneous tissue. The cadaver skin, which lacked the elasticity and coloring typical of a living subject, offered minimal resistance. The incision was made along predefined anatomical landmarks in order to replicate surgical precision despite the absence of bleeding (iliac crest, the lower edge of the gluteus maximus, and sagittal plane central to the gluteal region). Once the skin and subcutaneous tissue had been dissected and removed, the deep fascia of the gluteal region was exposed. Unlike living tissue, the fascia lacked elasticity and resilience. Nevertheless, the operators navigated it precisely, using blunt dissection and careful retraction with forceps to expose the underlying muscle planes.

Working in tandem, the gluteal muscles were separated. The surgeons meticulously separated the fibers of the gluteal muscles - the gluteus maximus, gluteus medius, and piriformis. The cadaver muscles were easier to manipulate, lacking contraction and tone. Careful dissection ensured that muscle integrity was maintained and anatomical boundaries were delineated.

The dissectors were careful in identifying the SN. Careful attention was paid to identifying the critical anatomical structure of the SN, which runs through the gluteal region and posterior thigh. Gentle manipulation exposed the nerve and the piriformis muscle. Given the lack of physiological response, the cadaveric SN offered minimal resistance to manipulation.

Finally, the PM and SN were fully exposed with meticulous dissection and retraction. The cadaveric tissues, devoid of vital signs, provided a static view of anatomical relationships. Any remaining connective tissue or fascial attachments were carefully dissected and removed to ensure complete exposure of both the PM and SN.

Consent for the study on the self-donated bodies was obtained from the same donors before their death. The study protocol conformed to the tenets of the Declaration of Helsinki.

## Discussion

Nowadays, the impact of anatomical variability on the success of various surgical procedures cannot be overstated. It is important to bear in mind that variations can be present in all the body regions and in organs that are located in these regions together with the close relationship with vessels in order to avoid vascular surgery complications [28,29]. 

These variations pose significant challenges to surgeons and, if not correctly recognized and managed, can lead to technical errors with unwanted iatrogenic consequences [30]. Even experienced surgeons may encounter difficulties when confronted with unexpected anatomical configurations during surgery, highlighting the importance of comprehensive and in-depth anatomical knowledge.

Failure to recognize variant anatomy is one of the most commonly cited technical errors leading to surgical injury [31]. These errors compromise patient safety and expose healthcare providers to malpractice claims, further emphasizing the need for improved technical competence in surgical practice.

Addressing this gap in technical skills requires a multifaceted approach: indeed, in these last years, 3D virtual technologies deeply contributed to anatomical education, even if the most attention should be payed to direct dissection in order to improve and refine the future physician's knowledge by the anatomical teaching “gold standard” [32-35]. Anatomy dissection courses tailored to surgical trainees, along with other supplementary material, remain a critical step in improving their understanding of the variability of human morphology [36,37]. By providing hands-on experience in dissecting cadaveric specimens, trainees can develop a more nuanced appreciation of anatomical variation and its implications for surgical procedures.

In addition, the incorporation of preoperative imaging techniques, such as magnetic resonance imaging (MRI) and computed tomography (CT) scans, can help to verify anatomical morphology prior to surgery [38,39]. This proactive approach allows surgeons to anticipate and plan for variations, reducing the likelihood of intraoperative surprises and associated complications.

The implementation of these preemptive interventions will provide significant benefits to healthcare systems. Improved surgical outcomes resulting from a better understanding of anatomical variability and improved preoperative planning can lead to reduced reoperation rates and increased patient satisfaction [31]. Furthermore, by reducing the incidence of surgical errors, such interventions will help to reduce the financial burden on healthcare providers associated with medical malpractice claims.

Finally, focusing on sciatica, a correct diagnosis through various imaging modalities can help the healthcare provider avoid invasive, useless, and harmful interventions [40]. Indeed, an in-depth understanding of the possible SN pattern within the PM interactions may allow physicians to choose surgical approaches or opt for nerve mobilization and muscle release [41,42].

It is worth noticing that the present study has some limitations, such as the small sample size. In addition, another limitation is the lack of knowledge of the donor's medical history, which could perhaps help to better understand the presumed presence of pain and its associated management. However, the observed case reports underline again that surgical outcomes and patients’ health require a concerted effort of education, training, and technological advancement in order to address the impact of anatomical variability. Moreover, concerning the existing literature on SN and PM variability, our findings on the dissector table underline the need to point attention also at the level of SN branching in TN and CPN.

Thus, by equipping surgical trainees with the necessary knowledge and skills to navigate anatomical complexity, and by using advanced imaging modalities to improve preoperative planning, healthcare systems can strive for safer and more effective surgical care, ultimately benefiting both patients and providers.

## Conclusions

Taking into account the above considerations, we can conclude by reiterating the immense importance of understanding anatomical variations for the successful outcome of a variety of diagnostic and putative surgical interventions. Negligence and lack of recognition of variable anatomy have led to frequent iatrogenic effects: such a gap should be filled to avoid adverse effects, injuries to patients, and malpractice claims. Anatomy dissection courses, focusing not only on normal anatomy and teaching surgical trainees but especially on human morphological "anomalies," are essential for physicians and surgeons, always together with the preoperative imaging techniques they need to confirm morphology. All of this knowledge can have a positive impact on patients, minimizing misdiagnosis and harmful interventions, thus improving not only patient satisfaction but also the healthcare system.
